# Computational Analysis of Single Nucleotide Polymorphisms Associated with Altered Drug Responsiveness in Type 2 Diabetes

**DOI:** 10.3390/ijms17071008

**Published:** 2016-06-25

**Authors:** Valerio Costa, Antonio Federico, Carla Pollastro, Carmela Ziviello, Simona Cataldi, Pietro Formisano, Alfredo Ciccodicola

**Affiliations:** 1 Institute of Genetics and Biophysics “Adriano Buzzati-Traverso”, National Research Council, Via Pietro Castellino 111, 80131 Naples, Italy; valerio.costa@igb.cnr.it (V.C.); antonio.federico@igb.cnr.it (A.F.); carla.pollastro@igb.cnr.it (C.P.); ziviello@gmail.com (C.Z.); simona.cataldi@igb.cnr.it (S.C.); 2 DiST, Department of Science and Technology, University of Naples “Parthenope”, 80134 Naples, Italy; 3 Department of Translational Medical Sciences, University of Naples “Federico II”, 80131 Naples, Italy; pietro.formisano@unina.it

**Keywords:** type 2 diabetes, drug responsiveness, computational predictions, pharmacogenomics, personalized medicine, RNA-sequencing

## Abstract

Type 2 diabetes (T2D) is one of the most frequent mortality causes in western countries, with rapidly increasing prevalence. Anti-diabetic drugs are the first therapeutic approach, although many patients develop drug resistance. Most drug responsiveness variability can be explained by genetic causes. Inter-individual variability is principally due to single nucleotide polymorphisms, and differential drug responsiveness has been correlated to alteration in genes involved in drug metabolism (*CYP2C9*) or insulin signaling (*IRS1*, *ABCC8*, *KCNJ11* and *PPARG*). However, most genome-wide association studies did not provide clues about the contribution of DNA variations to impaired drug responsiveness. Thus, characterizing T2D drug responsiveness variants is needed to guide clinicians toward tailored therapeutic approaches. Here, we extensively investigated polymorphisms associated with altered drug response in T2D, predicting their effects *in silico*. Combining different computational approaches, we focused on the expression pattern of genes correlated to drug resistance and inferred evolutionary conservation of polymorphic residues, computationally predicting the biochemical properties of polymorphic proteins. Using RNA-Sequencing followed by targeted validation, we identified and experimentally confirmed that two nucleotide variations in the *CAPN10* gene—currently annotated as intronic—fall within two new transcripts in this *locus*. Additionally, we found that a Single Nucleotide Polymorphism (SNP), currently reported as intergenic, maps to the intron of a new transcript, harboring *CAPN10* and *GPR35* genes, which undergoes non-sense mediated decay. Finally, we analyzed variants that fall into non-coding regulatory regions of yet underestimated functional significance, predicting that some of them can potentially affect gene expression and/or post-transcriptional regulation of mRNAs affecting the splicing.

## 1. Introduction

Diabetes is one of the leading causes of mortality in contemporary society [1]. A recent report by the International Diabetes Federation indicated an onset rate of about 8.4% in adults and a total number of 382 million cases of diabetes worldwide. This number is estimated to critically increase to up to 592 million by 2035 [1]. Thus, the World Health Organization (WHO) defines this phenomenon as a “global outbreak” [2]. Type 2 Diabetes (T2D), known as “non insulin-dependent diabetes”, is the most frequent form (with later onset) and occurs because of insulin defective function [3]. T2D affects more than 5% of the population in western countries and its spread is still increasing.

Diabetes is a chronic disease that over time leads to cardiovascular and blood vessel damage, neuro-, nephro- and retinopathy, with a dramatic impact on health, and high costs for National Health Systems [2]. Intensive programs focused on reducing lifestyle-derived risk factors for T2D have demonstrated substantial efficacy in lowering diabetes incidence in at-risk individuals [4]. The first step in T2D treatment, after the failure of lifestyle interventions, is the assumption of oral anti-diabetic drugs (OAD), such as sulphonylureas, metformin and thiazolidinediones (TZD). Sulphonylureas are insulin secretagogues that bind to an ATP-sensitive potassium channels. The most frequently used sulphonylureas in T2D are glibenclamide, gliclazide, glipizide and glimipiride but, in all cases, on a temporal long-scale of assumption, sulphonylureas decrease their effectiveness. Thus, it is recommended to keep the assumption in mind combined with other drugs such as metformin, decreasing insulin resistance in this way [5]. Metformin is a biguanide used in T2D to decrease hyperglycemia by suppressing glucose production by the liver [6]. Thiazolidinediones act mostly through the PPARs (Peroxisome Proliferator-Activated Receptors), whose endogenous ligands are fatty acids and eicosanoids [7]. Most T2D patients, after a long-term assumption, become less sensitive to drug treatment. Such a phenomenon has recently been associated with individual genomic variability. The introduction and wide diffusion of Genome-Wide Association Studies (GWAS) and of innovative technologies for high-throughput sequencing (Next Generation Sequencing, NGS) has turned pharmacogenetics into pharmacogenomics, shifting the interest from individual candidate genes to a genome-wide scale [8]. Indeed, pharmacogenetics, which focuses on hereditary genetic differences in drug metabolism, has gradually been sided with, and, in most cases, integrated by the pharmacogenomics that take inter- and intra-individual variation in gene expression and gene/proteins interaction networks into account. In this *scenario*, a growing number of studies have shown the influence of Single Nucleotide Polymorphisms (SNPs) on gene expression variation among populations [9,10], confirming that genome-wide and whole-transcriptome studies by NGS are fundamental to investigate complex traits/diseases, including T2D [11]. Therefore, as pharmacogenomics aims to provide the optimal treatment starting from the genetic and molecular etiology of the disease [12], T2D is an ideal candidate disease [3]. Indeed, it is characterized by the variable response of patients to drugs. Notably, although non-genetic factors—affecting hepatic, renal and intestinal functions—can influence OAD pharmacodynamics, the variable drug responsiveness can be partly explained by genetic factors. Indeed, SNPs in genes that are directly- or indirectly-involved in the metabolism, tropism and activity of drugs are often associated with altered drug responsiveness [12]. Thus, a comprehensive characterization of these genetic variants is crucial for diabetes research to guide clinicians toward better therapeutic approaches [13].

In this paper, we describe a systematic analysis of the most common SNPs associated with altered drug responsiveness in T2D, using both in silico and experimental approaches. The former were used to computationally predict the evolutionary conservation of polymorphic sites as well as the biochemical properties of the altered proteins (for SNPs falling in protein coding regions, termed coding SNPs or cSNPs). We also analyzed the genomic context that surrounds all the SNPs, searching for their proximity to CpG islands, transcription factors' binding sites or to other epigenetically relevant regions. We also experimentally addressed using RNA-Seq and targeted validations that some SNPs, previously annotated as intergenic or intronic, map to expressed regions. Our study predicts that some nucleotide variants—associated with reduced drug responsiveness in T2D patients—may affect protein functionality and suggests that some of them may act through still unexplored mechanisms, which arise from their coding potential.

## 2. Results

Several reports have so far described the association of SNPs and altered drug responsiveness in T2D [13]. These SNPs fall within genes with a clear relation to drug transport (*SLC22A1* and *SLC22A2*), metabolism (*CYP2C9*), activity (*PPARG* and *ABCC8*), genes that have a direct role in diabetes onset or progression (*KCNJ11*, *IRS1* and *TCF7L2*) or that have been frequently associated by GWAS to T2D and drug response (*CAPN10*), despite the fact that the exact mechanisms are still unclear. Here, a literature-based selection of SNPs in the most commonly associated genes has been performed, and the related SNPs have been analyzed using a combination of in silico and experimental approaches. Protein alignments and evolutionary conservation of polymorphic sites, analysis of protein tertiary structure (if available) and Sorting Intolerant From Tolerant (SIFT)/PolyPhen scores were used to predict the impact of protein-coding SNPs (cSNPs) on protein functionality. The most significant results are shown in the manuscript, and other data are collected in the Figures S1–S9. As many SNPs fall outside the coding regions of genes, we systematically searched CpG islands, transcription factors’ binding sites and relevant epimarks that can potentially be altered by SNPs. Using RNA-Seq on HEK293 cell lines, and taking advantage of a large collection of RNA-Seq datasets from different tissues and cell lines available in our laboratory, we also correlated SNPs to gene expression. Selected SNPs, their genomic localization, the possible alleles at that position, their IDs (according to the database of Single Nucleotide Polymorphisms, dbSNP), as well as the amino acid change (if any), are reported in Table 1.

### 2.1. Computational Predictions for Coding SNPs

**Figure 1:**
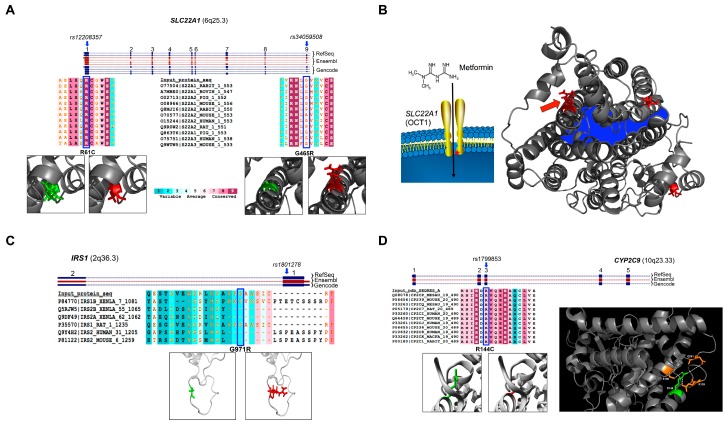
*SLC22A1* polymorphisms associated with metformin response. In the **upper** part, are schematically reported the genomic localizations of the SNPs analyzed in *SLC22A1*, *IRS1* and *CYP2C9* gene, in the panels (**A**), (**C**) and (**D**), respectively. RefSeq, Ensembl and GENCODE annotations are shown in different colors and are indicated in the Figure. The boxes indicate the exons and numbering is indicated above. The blue arrows indicate the genomic localization of the polymorphisms. In the **lower** part of panels (**A**), (**C**) and (**D**) are shown the 3D representations of amino acid changes corresponding to these SNPs. The canonical amino acid is reported in green and the mutated residue is shown in red. The evolutionary conservation score for each altered amino acid residue—according to Consurf software—is also shown. The specific amino acid is highlighted by a blue box; (**B**) on the **left** part, a schematic representation of the Oct1 protein in the cell membrane is shown. The red asterisk indicates the approximate position of the amino acid 465G, whose alteration is associated with reduced metformin responsiveness. On the **right** part of panel (**B**), the 3D protein structure of the entire Oct1 protein is shown. The **inner** portion of the channel is indicated in blue. Amino acid changes under evaluation are indicated in red (red arrow indicates G465R substitution); (**D**) on the **right**, the 3D protein structure of Cyp2c9 is shown, with the canonical R144 residue (in green) and three amino acids that are predicted to interact with it (in orange). Dashed white lines indicate the H-bonds among these residues.

For the *SLC22A1* gene, which encodes the Oct1 protein, results from the analysis of two cSNPs (rs12208357 and rs34059508) are discussed here. The analysis of rs34130495 is reported in the Figure S1. The rs12208357—a C/T substitution in the first exon of *SLC22A1* (Figure 1A)—causes R61C amino acid substitution into an extracellular topological domain of Oct1 protein (organic cation transporter 1; UniProt ID O15245) involved in the binding of the substrate. It leads to a decreased binding affinity to substrates [14]. Notably, the mutated residue is not charged, is smaller and more hydrophobic than the arginine, and it is predicted to alter inter-residual interactions and the correct folding of the ion channel. Protein alignment and analysis of evolutionary conservation revealed that arginine is highly conserved at this position in many species (Figure 1A). SIFT and PolyPhen scores (Table 2) indicate this SNP as damaging to protein functionality. The rs34059508 in exon 9 causes G465R amino acid change. Interestingly, 3D structure analysis of Oct1 protein revealed that G465 is close to the inner surface of the channel (Figure 1B), and the presence of a charged residue (arginine) in that position is likely to affect ion channel functionality. Accordingly, to support this observation, SIFT and PolyPhen scores were 0 and 0.999, respectively, indicating this SNP as “strongly damaging”.

However, we found at least three protein isoforms of Oct1 in primates that carry this amino acid substitution (Figure 1A), indicating that the polymorphic protein may be evolutionarily conserved. In addition, taking advantage of public human transcriptome data (available at Gene Expression Atlas; [15]) we found that *SLC22A1* has high tissue-specific expression, mainly restricted to the liver (Figure S10).

For the *IRS1* gene, we focused on a cSNP (rs1801278) in the exon 1 that causes the G971R change. Notably, it is often referred to as G972R, even though—according to dbSNP and UniProt databases—the exact nomenclature is G971R. Thus, we will refer to it as “G971R”. Protein alignment revealed that several species (reptiles and birds) carry this amino acid substitution (Figure 1C). Despite this substitution being conserved along the evolution, the biochemical properties of the residues are very different, as they differ in size and charge. The steric effect and the presence of a positive charge in the side chain are predicted to affect protein folding locally and/or the binding to other proteins.

In addition, PolyPhen marked this cSNP as damaging (Table 2). However, the absence of crystallographic data and of homologous proteins as templates did not permit us to determine a reliable 3D model to analyze such predicted damaging changes. Furthermore, analysis of public databases revealed *IRS1* to be almost ubiquitously expressed (Figure S10). This is in line with the pleiotropic role of Irs1 protein in the activation of the PI3K/AKT1/GSK3 signaling pathway, and, consequently, in the stimulation of glucose transport and of glycogen synthesis [16].

Finally, we analyzed cSNPs (rs1799853 and rs1057910) in the *CYP2C9* gene associated with sulphonylureas resistance. The gene encodes for cytochrome P450-2C9, a hepatic enzyme involved in the metabolism of most of sulphonylureas. The rs1799853 is a C/T variation in the exon 3 (R144C; Figure 1C) and is associated with resistance to sulphamethaxazole in T2D patients [17,18]. Although such a change is conserved not only in primates through evolution, the novel residue differs from the canonical for several characteristics. Indeed, the positive charge and the increased steric effect of the wild-type amino acid (arginine) with a neutral and smaller residue (cysteine) are predicted to have structural effects in the *core* of the protein. Computational prediction performed using the HOPE (Have (y)Our Protein Explained) prediction tool revealed that R144 forms hydrogen bonds with R139, S180 and Q261 (Figure 1C). Conversely, the mutated residue (i.e., C144) does not. Accordingly, both PolyPhen and SIFT scores indicate this cSNP as damaging (Table 2). *CYP2C9* gene has a peculiar expression pattern, mostly restricted to the liver, small intestine and duodenum (Figure S10). This is in line with the notion that this enzyme is responsible for sulphonylureas’ metabolism [18].

Similar conservation- and modeling-based approaches were also used to analyze SNPs in the coding regions of other genes commonly associated with altered drug response in T2D, such as *SLC22A2,* *KCNJ11, ABCC8* and *PPARG* genes. We could not in silico find any significant alteration in protein functionality according to the amino acid changes in these genes, or these nucleotide variations are already well characterized. A summary of the results is reported in Figures S1–S9.

### 2.2. SNPs Map to Regulatory Regions and Motifs of Transcription Factors

**Figure 2:**
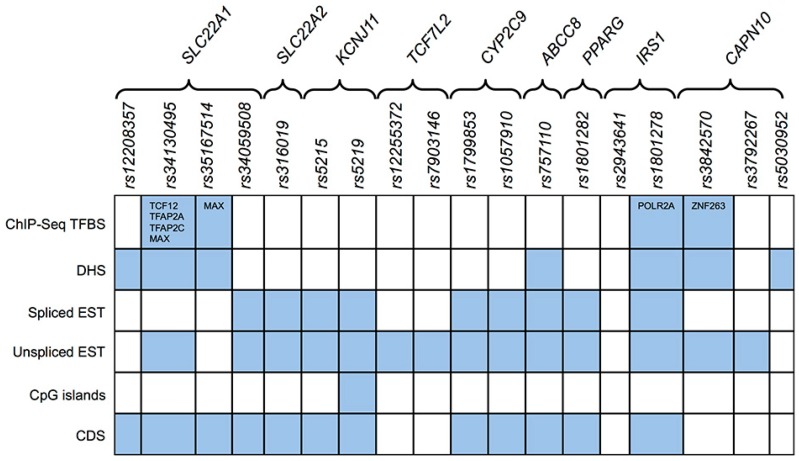
SNPs in drug resistance genes map to different regulatory regions. The panel shows a summary of the genomic regulatory elements that surround the SNPs herein analyzed. The cells in light blue indicate the presence of the SNP within a given regulatory element or genomic feature, whereas white cells indicate that the SNP does not fall within any of the analyzed regions. In the light blue cells of Transcription Factors Binding Sites detected by ChIP-Seq (ChIP-Seq TFBS row), the transcription factors are indicated that have binding sites potentially affected by the presence of the SNP (columns); DHS (DNAse Hypersensitive Sites); EST (Expressed Sequence Tag); CDS (Coding Sequences).

To address how the above-described SNPs may contribute to drug resistance, we extensively analyzed whether all variants reported in Table 1 map to regulatory sites, including transcription factors’ binding sites (TFBS), CpG islands, ESE/ESS (Exonic Splicing Enhancer/Exonic Splicing Silencer) and ISE/ISS (Intronic Splicing Enhancer/Intronic Splicing Silencer), and other regulatory regions (Figure 2; Table S1).

Taking advantage of public ChIP-Seq data of the ENCODE Consortium for several transcription factors (TFs) in different cell lines (Methods), we first assessed whether SNPs can disrupt confirmed TF binding sites. Interestingly, as schematized in Figure 2, rs34130495 (in the *SLC22A1* gene) disrupts the *consensus* sequence recognized by Tcf12, Tfap2a, Tfap2c and Max, and the rs35167514 in the same gene alters a binding site for Max, a master transcriptional regulator. The SNP rs1801278 (*IRS1* gene) falls within a Pol2a *consensus* sequence, while the rs3842570 in *CAPN10* gene falls within the binding site of Znf263, a negative transcriptional regulator.

Using a similar approach, we also analyzed whether drug resistance-associated SNPs map to genomic regions hypersensitive to DNAseI digestion, i.e., nucleosome-free transcriptional regulatory regions usually accessible to transcription factors. In line with the previous analysis, the rs34130495 and rs35167514 SNPs in *SLC22A1* falls within a strong DNAseI hypersensitive site (DHS), supported by experimental evidence in 80 cell lines. Similarly, the rs1801278 in the *IRS1* gene falls in DHS found in more than 90 cell lines. Weaker evidence has been also found for rs5030952 in *CAPN10* and rs757110 in *ABCC8* genes (DHS reported in 23 and 14 cell lines, respectively). The rs12208357 in *SLC22A1* gene falls in a DHS were reported only in hepatocytes and medulloblastoma, while, for the SNP rs3842570, in chorion cells and pancreatic islets.

Similar analysis was also carried out on computationally predicted *consensus* for TFBS (Jaspar and TRANSFAC databases). The rs5219 SNP—which causes the K23E substitution in *KCNJ11* gene (Figure S3)—maps to the *consensus* of the nuclear receptor PPARγ, and it is predicted to alter the binding affinity of this TF, a crucial player in glucose and lipid homeostasis and that is associated with metabolic disorders [19]. Notably, the same SNP also falls in a CpG island. Schematic summary of such information for other SNPs are reported in Table S1.

Finally, we investigated if drug resistance-associated SNPs may possibly affect the splicing extensively searching—in exons and introns—the regulatory splicing sites (ISE/ISS and ESE/ESS). This analysis revealed that only the rs3842570 and the rs12208357 in *CAPN10* and *SLC22A1* genes are located in an ISE. In particular, these *indels* were predicted to abrogate two binding sites for the same splicing factor, the Mbnl1 protein (AGCTGGG).

### 2.3. RNA-Seq Identifies New Transcripts of the CAPN10 Gene

**Figure 3:**
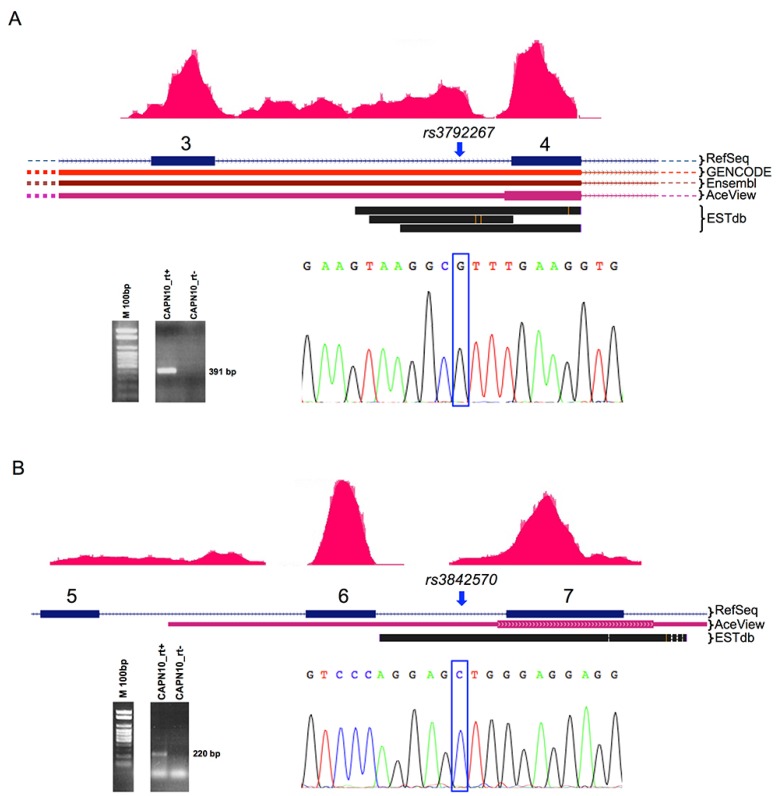
Schematic representation of the newly identified transcripts within the *CAPN10* gene *locus*. Genomic localization of the SNPs rs3842570 (**A**) and rs3792267 (**B**) in the *CAPN10* gene are shown. In the **upper** part of each panel: approximate localization of the SNPs according to RefSeq, Ensembl, Aceview, GENCODE and ESTdb annotations (boxes indicate the exons, whose numbers are shown above). The blue arrows indicate the genomic localization of the polymorphisms. Red peaks on gene annotations represent the reads’ coverage from the RNA-Seq experiment carried out on the HEK293 cell line. Peaks are indicative of active gene transcription. Agarose gel electrophoresis pictures for RT^+^ (Reverse Transcriptase) and RT-PCR assays in the HEK293 cell line are also reported for each panel. Blue boxes in the electropherograms (Sanger sequencing output) indicate the polymorphic site, which falls within a transcribed region.

**Figure 4:**
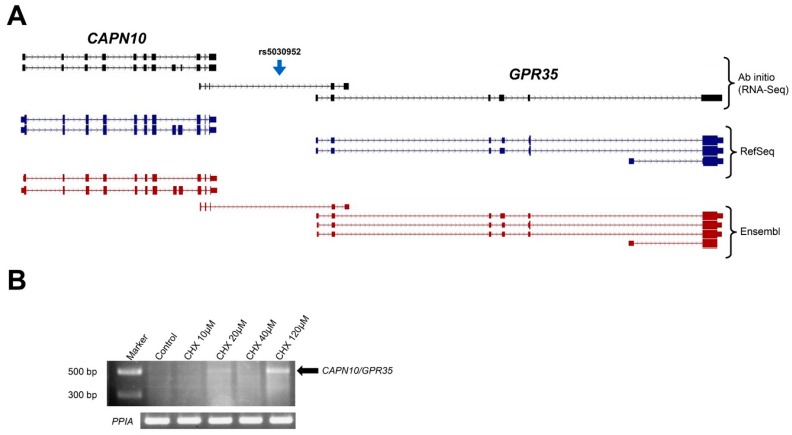
Schematic localization of *CAPN10/GPR35* *loci*. (**A**) Genomic organization of *CAPN10* (**left**) and *GPR35* (**right**) genes according to RefSeq and Ensembl annotations. The annotation shown in black represents the ab initio transcriptome of HEK293 cells as reconstructed from RNA-Seq data. Boxes indicate the exons. The blue arrow indicates the genomic localization of the polymorphism. A new transcript, automatically annotated in Ensembl database, links the two genes; (**B**) agarose gel electrophoresis picture in HEK293 cell line treated (or not) with cycloheximide (CHX) at growing concentrations as indicated above the gel image. The newly described transcript is indicated by the black arrow. *PPIA* gene, shown below, was used as a housekeeping control gene.

As gene annotations are rapidly being updated thanks to high-throughput sequencing, we also searched if these SNPs overlap newly annotated transcripts (both coding and not) and/or Expressed Sequence Tags (ESTs). Details for all analyzed SNPs are provided in the Table S1 and are graphically summarized in Figure 3 and Figure 4.

We used ab initio transcript reconstruction starting from RNA-Sequencing data, generated from HEK293 cell line sequencing. We found that the rs3842570 in *CAPN10*—described as intronic—maps within an actively transcribed region (Figure 3). The new transcript reconstructed from RNA-Seq overlaps an AceView [20] gene model (CAPN10 and GPR35.mAug10-unspliced) and EST BF528146. Similarly, the SNP rs3792267—located in the third intron of the same gene—overlaps a new transcribed region—unspliced ESTs (BQ899318, AL703891 and DA094595) from sciatic nerve and cerebellum libraries and a transcript annotated as “retained intron” in Ensembl and GENCODE databases (ENST00000494738). Taking advantage of RNA-Seq transcript reconstruction combined with a RT-PCR assay in RT^+^ (Reverse Transcriptase) and RT-cDNA from HEK293 cell line (Methods), we could confirm that both of the SNPs map to actively transcribed regions, despite being currently annotated as “intronic” SNPs of the *CAPN10* gene. Direct Sanger sequencing validated our findings (Figure 3).

In addition, RNA-Seq data revealed that the rs5030952, annotated as “intergenic” and associated with *CAPN10* gene, maps in the intron of two new transcripts spanning *CAPN10* and *GPR35* genes. Notably, one of these transcripts (details in Figure 4) is annotated in University of California Santa Cruz Genome Browser (UCSC Genome Browser), Ensembl and GENCODE databases as “non-sense mediated decay” (NMD).

RT-PCR, upon treatment of HEK293 cells with CHX, confirmed the presence of this new transcript generated by trans-splicing between *CAPN10* and *GPR35* genes, revealing that it undergoes NMD (Figure 4). We concluded that rs5030952 should be considered an ”intronic” SNP of this new transcript encompassing *CAPN10* and *GPR35 loci*.

Similarly, we found that the intronic SNPs associated with *TCF7L2* (rs12255372 and rs7903146) map to a transcribed region (data not shown) and overlap two unspliced ESTs (details in the Table S1).

Finally, taking advantage of our recently published—and still unpublished—RNA-Seq datasets, we investigated whether the presence of these SNPs may correlate to gene expression levels. RNA-Seq datasets of two HEK293 replicates, 22 thyroid [21], 2 MCF7 [22], eight heart biopsies (manuscript in preparation) were analyzed in order to find samples homozygous for the wild-type, heterozygous or homozygous for the alternative alleles. However, we could find a significant number of samples for each category only for the *KCNJ11*, *CAPN10* and *ABCC8* genes. No significant correlation could be found between SNPs in these genes and the mRNA levels of related genes (Figure S11).

## 3. Discussion

A comprehensive analysis of the most relevant single nucleotide variants associated with T2D predisposition and/or with altered drug responsiveness is crucial to understanding their potential contribution to the phenotype. Previous studies have highlighted the association between polymorphic variants and the onset of drug responsiveness in T2D patients. In this regard, our work aimed to systematically infer computational predictions about the effects of such common variations on protein functionality or about their potential impact on genomic regulatory sites. The combination of different computational approaches used herein has confirmed that the organic cation transporter family plays a pivotal role in drug responsiveness. Oct1 and Oct2 proteins are involved in the uptake of organic cations (including drugs) from the bloodstream into hepatocytes [23]. SNPs in the Oct1 encoding gene (*SLC22A1*) can influence drug response because of their ability to alter hepatic drug clearance as well as the uptake and elimination of drugs. This mechanism has been demonstrated for the 1-methy-4-phenyl-1,2,3,6-tetrahydropyridine (MPTP) [24]. Public RNA-Seq data confirmed the liver-specific expression of this gene, and our in silico prediction revealed that two *SLC22A1* cSNPs (Figure 1A) can potentially be damaging to protein functionality. These predictions strengthen GWAS data about the relationship between *SLC22A1* and metformin resistance in T2D. In addition, our 3D model of the polymorphic Oct1 protein has indicated that G465R amino acid change may influence metformin uptake since it is located in close proximity of the inner surface of Oct1 channel. Regarding the *CYP2C9* gene, it has been established that it plays an important role in the pharmacokinetics of sulphonylureas [25]. Arg144Cys substitution in the *CYP2C9* gene has been shown to markedly reduce the rates of substrate metabolism [26,27]. Specifically, carriers of polymorphic *CYP2C9* variants may have both higher therapeutic response and a higher incidence of hypoglycemic adverse events [28]. We have predicted that the lack of arginine in position 144 affects protein stability as this residue is critical in creating H-bonds that contact serine in position 180 and glutamine in position 261 (Figure 1C). It suggests a potential mechanism for the reduced activity of the mutated enzyme.

Whereas it is arguable that protein-coding SNPs can affect protein functionality—and then that they can associate to clinical manifestation of the disease—it is quite difficult to explain the contribution of intronic or intergenic SNPs. Indeed, most of analyses reported in literature for T2D have been performed on cSNPs. Here, we have considered nucleotide variations that potentially affect regulatory genomic elements since most of SNPs associated by GWAS to complex traits/diseases fall outside protein-coding regions. Therefore, a large fraction of these may determine the loss (or gain) of regulatory functions. Interestingly, we found that all analyzed SNPs fall within at least one TF binding site or genomic region overlapping a DHS and/or CpG island or a splicing regulatory region, according to public ChIP-Seq data and computational predictions. In particular, since few studies investigated the role of polymorphism rs5219 in the KCNJ11 gene in therapeutic response to sulphonylureas [9] or hypoglycemia risk [29], we inferred that this SNP falls in a PPARγ binding site and within a CpG island located in the gene body, possibly linking such variant to altered *KCNJ11* expression. However, although we could not find any statistically significant association between this SNP and mRNA levels in our RNA-Seq datasets, we cannot definitely exclude such a mechanism. More interestingly, we found that SNPs, until now annotated as “intronic” or “intergenic”, are misannotated. Indeed, through ab initio transcriptome reconstruction and experimental validations we have shown they map inside mature mRNAs.

Two intronic *CAPN10* SNPs (rs3842570 and rs3792267) belong to actively transcribed (and spliced) *CAPN10* transcripts. Our findings are in line with the gene predictions of AceView database and with ESTs. Notably, the former SNP (a 1 bp deletion) is located in an intron splicing enhancer region, and its presence is predicted to affect the binding of two different splicing factors. We cannot exclude that the presence of this SNP may account for splicing variations that give rise to the new mRNA isoform. Similarly, our data indicate that the “intergenic” rs5030952 falls in the intron of a new transcript, generated by trans-splicing between *CAPN10* and *GPR35* genes.

Despite being aware that computational predictions alone cannot prove any causal relation between SNPs and the clinical outcome in patients, we believe that combining different in silico approaches to experimental validations can provide insights to improving the knowledge underlying the SNPs’ effect on complex phenotypes. In particular, growing evidence indicates that determining specific genotypes is crucial to establishing targeted therapeutic strategies. Thus, it would be desirable to have a deeper understanding of the mechanisms by which environmental and epigenetic factors interact with patient genetic *milieu* [30], and in contrast, how SNPs are likely to modify genomic regions that are involved in the epigenetic regulation of gene expression or directly modify the primary sequence of non-coding RNAs, and potentially their function. In this *scenario*, our study highlights that even SNPs that do not affect the protein coding ability of a gene can potentially have a functional impact on splicing and/or gene expression, possibly through epigenetic mechanisms, or they may modify the *consensus* sequence of transcription factors. Functional targeted studies are needed to experimentally demonstrate the impact of such variations mapping in non-coding regions of the genome.

## 4. Materials and Methods

By extensive literature and database browsing, we selected 18 SNPs in nine genes commonly associated with altered responsiveness to pharmacotherapy of T2D [13,31,32,33]. SNPs have been categorized as “exonic”, “intronic” or “intergenic” according to RefSeq gene annotation (Table 1) [31].

### 4.1. Computational Predictions of Amino Acid Changes

Amino acidic sequences and functional protein domains of the annotated proteins were retrieved by the UniProt database [34]. The functional effects of nucleotide variants were evaluated only for cSNPs, using SIFT and PolyPhen scores, through the VEP tool (Variant Effect Predictor) in the Ensembl database [35].

The prediction of biochemical and structural effects of amino acidic substitutions was carried out using Project HOPE [36]. For some cSNPs, HOPE was unable to predict structural effects of the substitution, due to the lack of structural information in public databases. In these cases, the prediction of tridimensional structures of the polymorphic proteins was performed applying a homology modeling approach through the web server I-TASSER [37]. Obtained models were finally rendered using the PyMol system [38].

Evolutionary analysis of the residue changes induced by cSNPs was inferred using human and other vertebrates species. In particular, the conservation of polymorphic sites was investigated among vertebrates through the alignment of the longest sequence for the human annotated protein (UniProt) and orthologous protein sequences. These alignments were then submitted to the web server ConSurf [39]. Consurf-based analysis was launched using Swiss-Prot as target database (except for *ABCC8* for which we selected UniRef90) and running at least three iterations with the PSI-BLAST algorithm. For the remaining parameters, we used the default settings.

### 4.2. In Silico Analysis of Epimarks and Regulatory Motifs

The genomic coordinates and the allelic frequencies of selected SNPs have been downloaded from dbSNP (v138) database [40]. The table browser option of UCSC Genome Browser [41] was used to retrieve custom tracks of interest (ENCODE Transcription Factor ChIP-seq Uniform Peaks, ENCODE DNaseI Hypersensitive Site Master List). To investigate if SNPs may alter splicing sites, or related regulatory regions such as exonic and/or intronic splicing enhancer/silencer (ESE/ESS and ISE/ISS) we used the web tool RegRNA 2.0 [42], as previously described in Scarpato et al. [43]. Briefly, FASTA files of the nucleotide sequences surrounding the polymorphic site (10 bp upstream and 10 bp downstream the SNP) were retrieved by the UCSC Genome Browser and submitted to RegRNA web server. The analysis of correlation between gene expression and SNPs has been carried out using our published [21,22] and unpublished RNA-Seq data. Specifically, such datasets were inspected using the Integrative Genomic Viewer (IGV) [44]. Expression profiles of the genes associated with reduced drug responsiveness has been retrieved from the RNA-Seq samples of 122 human individuals from 32 different tissues (E-MTAB-2836) available on the Gene Expression Atlas website (available at: http://www.ebi.ac.uk/) [45].

### 4.3. RNA-Sequencing: Library Preparation and Data Analysis

Briefly, RNA has been isolated and quantified from HEK293 cell lines as previously described in [46]. Paired-end libraries were prepared using the TruSeq RNA Sample Preparation Kit (Illumina, San Diego, CA, USA) and sequenced on the Illumina HiSeq2000 platform according to the manufacturer’s instructions. Reads mapping was performed using TopHat v.2.0.7 [47], and ab initio transcriptome reconstruction was carried out with Cufflinks [42], setting GENCODE v19 as reference transcriptome. Coverage files were generated using the *genomecoverageBed* function of BEDtools [48]. Visual inspection on the UCSC Genome Browser of coverage files revealed the presence of new transcripts overlapping the genomic coordinates of analyzed SNPs. PCR duplicates were removed using Picard tools v1.117 (Broad Institute, Cambridge, MA, USA) (available at: http://broadinstitute.github.io/picard/) and SNP calling was carried out using GATK v3.3 workflow (Broad Institute, Cambridge, MA, USA) optimized for RNA-Seq reads, as described in [49]. Normalization, filtering, data inspection and general data analysis statistics were performed using RNASeqGUI [50].

### 4.4. In Vitro Validations

The experimental validation for the new transcripts of *CAPN10* and for the *CAPN10/GPR35* transcript have been performed on the RNA isolated from HEK293 cell line. HEK293 cells were cultured in Dulbecco’s modified Eagle’s medium (DMEM, Life Technologies, Carlsbad, CA, USA) supplemented with 10% fetal bovine serum (FBS), glutamine (2 mmol/L), penicillin (100 units/mL) and streptomycin (100 units/mL) at 37 °C in a humidified atmosphere of 95% air and 5% CO_2_. Cells were treated with cycloheximide (CHX) at increasing doses (10, 20, 40, 120 µM) or with the vehicle control (DMSO) for 6 h as described in [51]. Total RNA was isolated from treated cells using TRIzol solution (Invitrogen, Waltham, MA, USA) according to the manufacturer’s instructions. RNA obtained by each samples was reverse transcribed using “high-capacity cDNA reverse-transcription kit” (Applied Biosystems, Foster City, CA, USA) and the cDNA were used as template for RT-PCR assays. PCR assays have been performed with MyTaq DNA Polimerase (Bioline Reagents Ltd., London, UK) using these condition: 95 °C for 3 min, followed by 40 cycles at 95 °C for 30 s, 58 °C for 30 s, 72 °C for 70 s, and 72 °C for 7 min. Sanger sequencing to confirm the presence of rs3842570 and rs3792267 in *CAPN10* has been carried on PCR amplicons carried out with 2.5U AmpliTaq-Gold (Life Technologies, Carlsbad, CA, USA) according to the manufacturer’s protocol. PCR amplicons were verified through electrophoresis on agarose gel (1.5%).

## Figures and Tables

**Table 1:** Type 2 diabetes (T2D) drug responsiveness associated Single Nucleotide Polymorphisms (SNPs).

Chromosome	Position	Localization	Gene	Alleles	SNP ID	Amino Acid Change
6	160543148	Exon	*SLC22A1*	C/T	rs12208357	R61C
	160560824	Exon		A/G	rs34130495	G401S
	160560881	Exon		-/A	rs35167514	420del
	160575837	Exon		A/C/G	rs34059508	G465R
6	160670282	Exon	*SLC22A2*	G/T	rs316019	S270A
11	17409572	Exon	*KCNJ11*	T/C	rs5219	K23E
	17408630	Exon		G/A	rs5215	V337I
10	114808902	Intron	*TCF7L2*	G/T	rs12255372	-
	114758349	Intron		C/T	rs7903146	-
10	96702047	Exon	*CYP2C9*	C/T	rs1799853	R144C
	96741053	Exon		A/C	rs1057910	I359L
11	17418477	Exon	*ABCC8*	G/T	rs757110	A1369S
3	12393125	Exon	*PPARG*	C/G	rs1801282	P12A
2	227093745	Intergenic	*IRS1*	C/T	rs2943641	-
	227660544	Exon		G/A	rs1801278	G971R
2	241534293	Intron	*CAPN10*	Indel	rs3842570	-
	241531174	Intron		A/G	rs3792267	-
	241542703	Intron		C/T	rs5030952	-

**Table 2:** Sorting Intolerant From Tolerant (SIFT) and PolyPhen scores and predictions for coding SNPs (cSNPs).

Gene	SNP	Codon	Amino Acid	Score		Prediction	
				SIFT	PolyPhen	SIFT	PolyPhen
*SLC22A1*	rs12208357	CGC/TGC	R61C	0.01	0.954	D	PD
	rs34130495	GGC/AGC	G401S	0	0.999	D	PD
	rs35167514	ATG/TG	M420del	-	-	-	-
	rs34059508	GGA/AGA	G465R	0	0.984	D	PD
*SLC22A2*	rs316019	TCT/GCT	S270A	0.01	0.524	D	PD
*KCNJ11*	rs5215	GTC/ATC	V337I	0.08	0.015	T	B
	rs5219	AAG/GAG	K23E	0.17	0	T	B
*CYP2C9*	rs1799853	CGT/TGT	R144C	0.03	0.978	D	PD
	rs1057910	ATT/CTT	I359L	0.09	0.45	T	PD
*ABCC8*	rs757110	GCC/TCC	A1369S	0.73	0	T	B
*PPARG*	rs1801282	CCA/GCA	P12A	0	0	D	B
*IRS1*	rs1801278	GGG/AGG	G971R	0.51	0.986	T	PD

D = deleterious; T = tolerated; PD = probably damaging; B = Benign.

## Supplementary Materials

Supplementary materials can be found at http://www.mdpi.com/1422-0067/17/7/1008/s1.

## References

[1] Shaw J.E., Sicree R.A., Zimmet P.Z. (2010). Global estimates of the prevalence of diabetes for 2010 and 2030. Diabetes Res. Clin. Pract..

[2] World Health Organization (WHO) (2016). Diabetes.

[3] Hattersley A., Bruining J., Shield J., Njolstad P., Donaghue K.C. (2009). The diagnosis and management of monogenic diabetes in children and adolescents. Pediatr. Diabetes.

[4] Mannino G.C., Sesti G. (2012). Individualized therapy for type 2 diabetes: Clinical implications of pharmacogenetic data. Mol. Diagn. Ther..

[5] Rendell M. (2004). The role of sulphonylureas in the management of type 2 diabetes mellitus. Drugs.

[6] Kirpichnikov D., McFarlane S.I., Sowers J.R. (2002). Metformin: An update. Ann. Intern. Med..

[7] Hauner H. (2002). The mode of action of thiazolidinediones. Diabetes Metab. Res. Rev..

[8] Becker M.L., Pearson E.R., Tkáč I. (2013). Pharmacogenetics of oral antidiabetic drugs. J. Endocrinol. Diabetes.

[9] Javorsky M., Klimcakova L., Schroner Z., Zidzik J., Babjakova E., Fabianova M., Kozarova M., Tkacova R., Salagovic J., Tkac I. (2012). *KCNJ11* gene E23K variant and therapeutic response to sulphonylureas. Eur. J. Intern. Med..

[10] He Y.Y., Zhang R., Shao X.Y., Hu C., Wang C.R., Lu J.X., Bao Y.Q., Jia W.P., Xiang K.S. (2008). Association of *KCNJ11* and *ABCC8* genetic polymorphisms with response to repaglinide in Chinese diabetic patients. Acta Pharmacol. Sin..

[11] Montgomery S.B., Sammeth M., Gutierrez-Arcelus M., Lach R.P., Ingle C., Nisbett J., Guigo R., Dermitzakis E.T. (2010). Transcriptome genetics using second generation sequencing in a Caucasian population. Nature.

[12] Emilien G., Ponchon M., Caldas C., Isacson O., Maloteaux J.M. (2000). Impact of genomics on drug discovery and clinical medicine. QJM.

[13] Di Stefano J.K., Watanabe R.M. (2010). Pharmacogenetics of Anti-Diabetes Drugs. Pharmaceuticals.

[14] Pruitt K.D., Brown G.R., Hiatt S.M., Thibaud-Nissen F., Astashyn A., Ermolaeva O., Farrell C.M., Hart J., Landrum M.J., McGarvey K.M. (2014). RefSeq: An update on mammalian reference sequences. Nucleic Acids Res..

[15] Petryszak R., Burdett T., Fiorelli B., Fonseca N.A., Gonzalez-Porta M., Hastings E., Huber W., Jupp S., Keays M., Kryvych N. (2014). Expression Atlas update—A database of gene and transcript expression from microarray- and sequencing-based functional genomics experiments. Nucleic Acids Res..

[16] Aquilante C.L. (2010). Sulfonylurea pharmacogenomics in Type 2 diabetes: The influence of drug target and diabetes risk polymorphisms. Expert Rev. Cardiovasc. Ther..

[17] Kirchheiner J., Brockmöller J. (2005). Clinical consequences of cytochrome P450 2C9 polymorphisms. Clin. Pharmacol. Ther..

[18] Wen X., Wang J.S., Backman J.T., Laitila J., Neuvonen P.J. (2002). Trimethoprim and sulfamethoxazole are selective inhibitors of CYP2C8 and CYP2C9, respectively. Drug Metab. Dispos..

[19] Florez J.C., Jablonski K.A., Sun M.W., Bayley N., Kahn S.E., Shamoon H., Hamman R.F., Knowler W.C., Nathan D.M., Altshuler D. (2007). Effects of the type 2 diabetes-associated *PPARG* P12A polymorphism on progression to diabetes and response to troglitazone. J. Clin. Endocrinol. Metab..

[20] Thierry-Mieg D., Thierry-Mieg J. (2006). AceView: A comprehensive cDNA-supported gene and transcripts annotation. Genome Biol..

[21] Costa V., Esposito R., Pallante P., Ciccodicola A., Fusco A. (2015). The “next-generation” knowledge of papillary thyroid carcinoma. Cell Cycle.

[22] Aversa R., Sorrentino A., Esposito R., Ambrosio M.R., Amato A., Zambelli A., Ciccodicola A., D’Apice L., Costa V. (2016). Alternative Splicing in Adhesion- and Motility-Related Genes in Breast Cancer. Int. J. Mol. Sci..

[23] Shikata E., Yamamoto R., Takane H., Shigemasa C., Ikeda T., Otsubo K., Ieiri I. (2007). Human organic cation transporter (OCT1 and OCT2) gene polymorphisms and therapeutic effects of metformin. J. Hum. Genet..

[24] Ekeruo I.A., Solhpour A., Taegtmeyer H. (2013). Metformin in Diabetic Patients with Heart Failure: Safe and Effective?. Curr. Cardiovasc. Risk Rep..

[25] Klen J., Dolžan V., Janež A. (2014). *CYP2C9*, *KCNJ11* and *ABCC8* polymorphisms and the response to sulphonylurea treatment in type 2 diabetes patients. Eur. J. Clin. Pharmacol..

[26] Wei L., Locuson C.W., Tracy T.S. (2007). Polymorphic variants of CYP2C9: Mechanisms involved in reduced catalytic activity. Mol. Pharmacol..

[27] Crespi C.L., Miller V.P. (1997). The R144C change in the CYP2C9*2 allele alters interaction of the cytochrome P450 with NADPH: Cytochrome P450 oxidoreductase. Pharmacogenetics.

[28] Becker M.L., Visser L.E., Trienekens P.H., Hofman A., van Schaik R.H., Stricker B.H. (2008). Cytochrome P450 2C9 *2 and *3 polymorphisms and the dose and effect of sulfonylurea in type II diabetes mellitus. Clin. Pharmacol. Ther..

[29] Ragia G., Tavridou A., Petridis I., Manolopoulos V.G. (2012). Association of *KCNJ11* E23K gene polymorphism with hypoglycemia in sulfonylurea-treated type 2 diabetic patients. Diabetes Res. Clin. Pract..

[30] Raciti G.A., Nigro C., Longo M., Parrillo L., Miele C., Formisano P., Béguinot F. (2014). Personalized medicine and type 2 diabetes: Lesson from epigenetics. Epigenomics.

[31] Pollastro C., Ziviello C., Costa V., Ciccodicola A. (2015). Pharmacogenomics of Drug Response in Type 2 Diabetes: Toward the Definition of Tailored Therapies?. PPAR Res..

[32] Sakamoto Y., Inoue H., Keshavarz P., Miyawaki K., Yamaguchi Y., Moritani M., Kunika K., Nakamura N., Yoshikawa T., Yasui N. (2007). SNPs in the *KCNJ11-ABCC8* gene locus are associated with type 2 diabetes and blood pressure levels in the Japanese population. J. Hum. Genet..

[33] Song Y., Niu T., Manson J.E., Kwiatkowski D.J., Liu S. (2004). Are variants in the *CAPN10* gene related to risk of type 2 diabetes? A quantitative assessment of population and family-based association studies. Am. J. Hum. Genet..

[34] The UniProt Database. http://www.uniprot.org.

[35] McLaren W., Pritchard B., Rios D., Chen Y., Flicek P., Cunningham F. (2010). Deriving the consequences of genomic variants with the Ensembl API and SNP Effect Predictor. Bioinformatics.

[36] Venselaar H., Te Beek T.A., Kuipers R.K., Hekkelman M.L., Vriend G. (2010). Protein structure analysis of mutations causing inheritable diseases. An e-Science approach with life scientist friendly interfaces. BMC Bioinform..

[37] Zhang Y. (2008). I-TASSER server for protein 3D structure prediction. BMC Bioinform..

[38] The PyMOL Molecular Graphics System, Version 1.8 Schrödinger, LLC. http://www.pymol.org.

[39] Celniker G., Nimrod G., Ashkenazy H., Glaser F., Martz E., Mayrose I., Pupko T., Ben-Tal N. (2013). ConSurf: Using Evolutionary Data to Raise Testable Hypotheses about Protein Function. Isr. J. Chem..

[40] Sherry S.T., Ward M., Sirotkin K. (1999). dbSNP-database for single nucleotide polymorphisms and other classes of minor genetic variation. Genome Res..

[41] Kent W.J., Sugnet C.W., Furey T.S., Roskin K.M., Pringle T.H., Zahler A.M., Haussler D. (2002). The human genome browser at UCSC. Genome Res..

[42] Chang T.H., Huang H.Y., Hsu J.B., Weng S.L., Horng J.T., Huang H.D. (2013). An enhanced computational platform for investigating the roles of regulatory RNA and for identifying functional RNA motifs. BMC Bioinform..

[43] Scarpato M., Esposito R., Evangelista D., Aprile M., Ambrosio M.R., Angelini C., Ciccodicola A., Costa V. (2014). AnaLysis of Expression on human chromosome 21, ALE-HSA21: A pilot integrated web resource. Database.

[44] Thorvaldsdóttir H., Robinson J.T., Mesirov J.P. (2013). Integrative Genomics Viewer (IGV): High-performance genomics data visualization and exploration. Brief Bioinform..

[45] Kapushesky M., Emam I., Holloway E., Kurnosov P., Zorin A., Malone J., Rustici G., Williams E., Parkinson H., Brazma A. (2010). Gene expression atlas at the European bioinformatics institute. Nucleic Acids Res..

[46] Costa V., Casamassimi A., Roberto R., Gianfrancesco F., Matarazzo M.R., D’Urso M., D’Esposito M., Rocchi M., Ciccodicola A. (2009). DDX11L: A novel transcript family emerging from human subtelomeric regions. BMC Genom..

[47] Trapnell C., Pachter L., Salzberg S.L. (2009). TopHat: Discovering splice junctions with RNA-Seq. Bioinformatics.

[48] Quinlan A.R., Hall I.M. (2010). BEDTools: A flexible suite of utilities for comparing genomic features. Bioinformatics.

[49] Costa V., Esposito R., Ziviello C., Sepe R., Bim L.V., Cacciola N.A., Decaussin-Petrucci M., Pallante P., Fusco A., Ciccodicola A. (2015). New somatic mutations and *WNK1-B4GALNT3* gene fusion in papillary thyroid carcinoma. Oncotarget.

[50] Russo F., Angelini C. (2014). RNASeqGUI: A GUI for analysing RNA-Seq data. Bioinformatics.

[51] Dedman A.M., Majeed Y., Tumova S., Zeng F., Kumar B., Munsch C., Bateson A.N., Wittmann J., Jäck H.M., Porter K.E. (2011). TRPC1 transcript variants, inefficient nonsense-mediated decay and low up-frameshift-1 in vascular smooth muscle cells. BMC Mol. Biol..

