# Supplementary material for: Computational Analysis of Single Nucleotide Polymorphisms Associated with Altered Drug Responsiveness in Type 2 Diabetes
Source: Int J Mol Sci. 2016 Jun 25;17(7):1008. doi: 10.3390/ijms17071008 (PMC4964384; doi:10.3390/ijms17071008)
Supplement: Supplementary file 1 [file ijms-17-01008-s001.pdf]

Supplementary Materials: Computational Analysis of Single Nucleotide Polymorphisms Associated with Altered Drug Responsiveness in Type 2 Diabetes

Valerio Costa, Antonio Federico, Carla Pollastro, Carmela Ziviello, Simona Cataldi, Pietro Formisano and Alfredo Ciccodicola

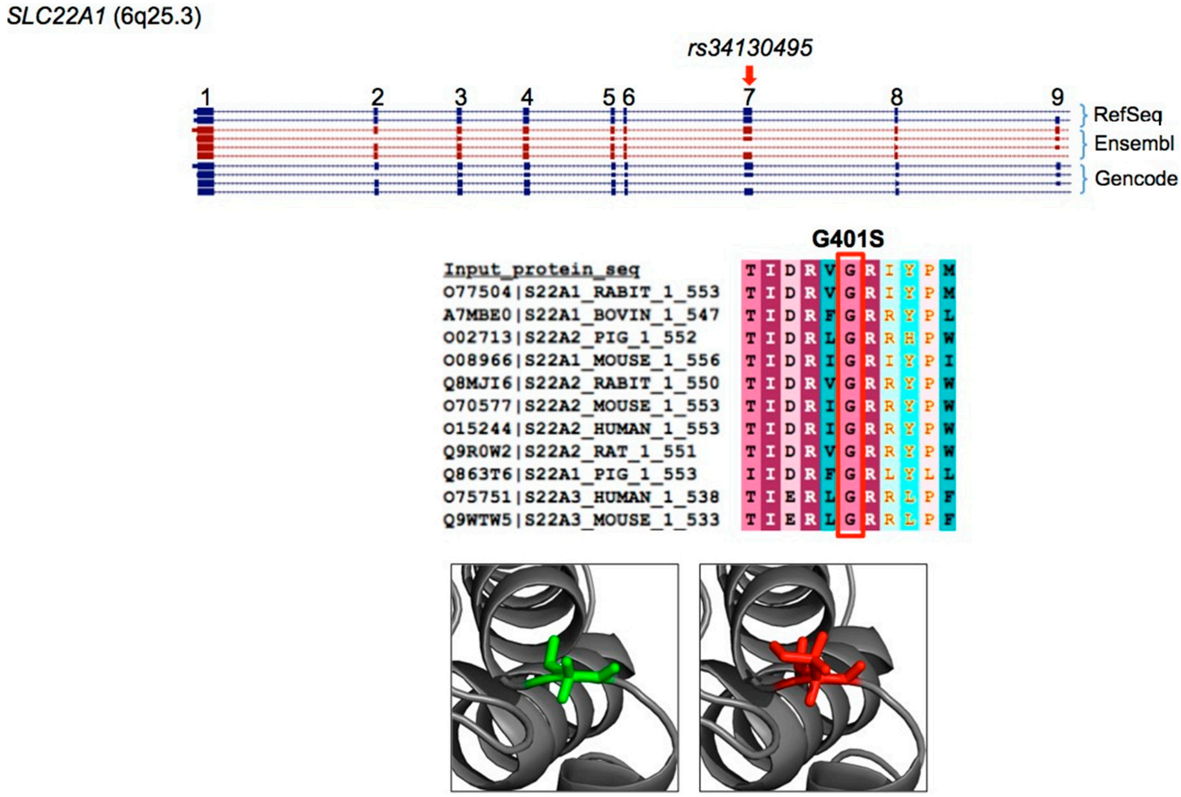

Figure S1. The polymorphism rs34130495 consists in a non-synonimous A/G substitution in the exon 7 which carries to a G to S amino acidic change in position 401. The SIFT and PolyPhen scores were calculated indicating it as potentially damaging. The glycine residue is highly conserved among vertebrates in that position indicated by red box. In addition, as glycine is the smallest residue and its presence confers a considerable flexibility to protein, this mutation is predicted to affect protein functionality by locally increasing the rigidity of protein structure. In the tridimensional protein structure, the wild-type residue is shown in green, the polymorphic residue in red.

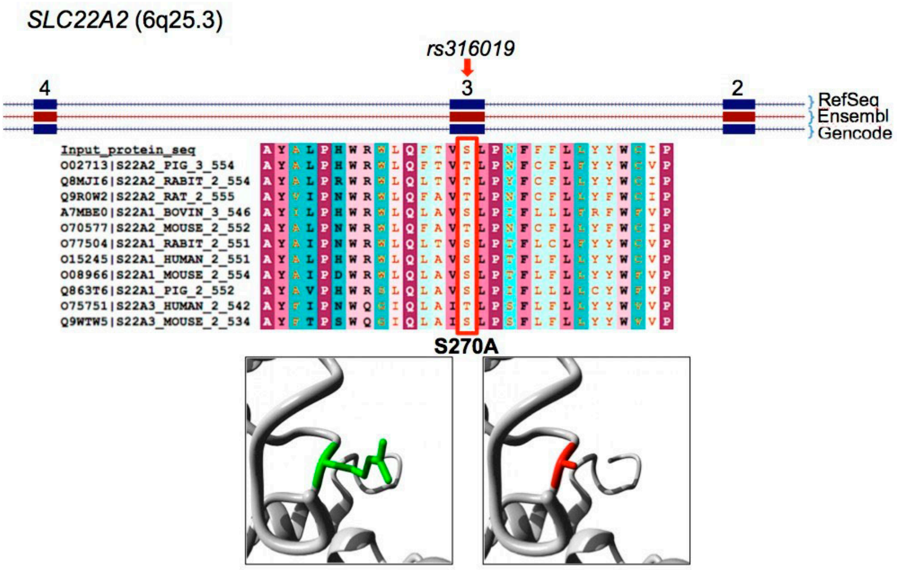

Figrue S2. This SNP-G/T change in the exon 3—determines a serine to alanine substitution in position 270 of Oct2 protein (UniProt ID O15244). These two amino acids significantly differ for charge and hydrophobicity. Given the high sequence similarity among the members of *SLC22A* gene family, Oct2 tertiary structure is predicted to be very similar to the one of Oct1. Indeed, according to UniProt annotation, the amino acid substitution falls within the sixth transmembrane helix. SIFT and PolyPhen scores indicate this SNP as "deleterious" and "probably damaging" to protein functionality, respectively. In the multiple sequence alignment, the red box indicates the polymorphic amino acid. In the tridimensional protein structure, the wild-type residue is shown in green, the polymorphic residue in red.

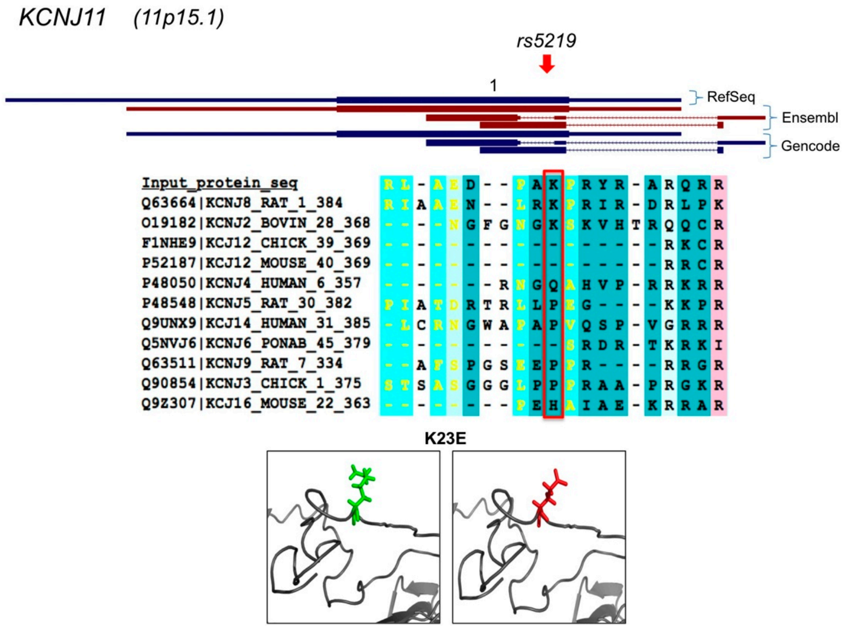

Figure S3. The polymorphism rs5219 falls in the coding sequence of *KCNJ11* gene (RefSeq NM_000525.3) and causes, at protein level (UniProt ID Q14654), a K to E amino acid change in position 23. This change is not conserved on evolutionary scale, but some orthologous sequences carry, at this position, amino acids with similar chemical and physical characteristics (red box). This indicates that such substitution is conservative. The most relevant differences between canonical and novel amino acid are in their size and charge. Indeed, lysine is bigger than glutammate, and they are differently charged. Despite these considerations, the evolutionary analysis and the SIFT and PolyPhen scores indicate that such substitution is likely to be benign. In the tridimensional protein structure, the wild-type residue is shown in green, the polymorphic residue in red.

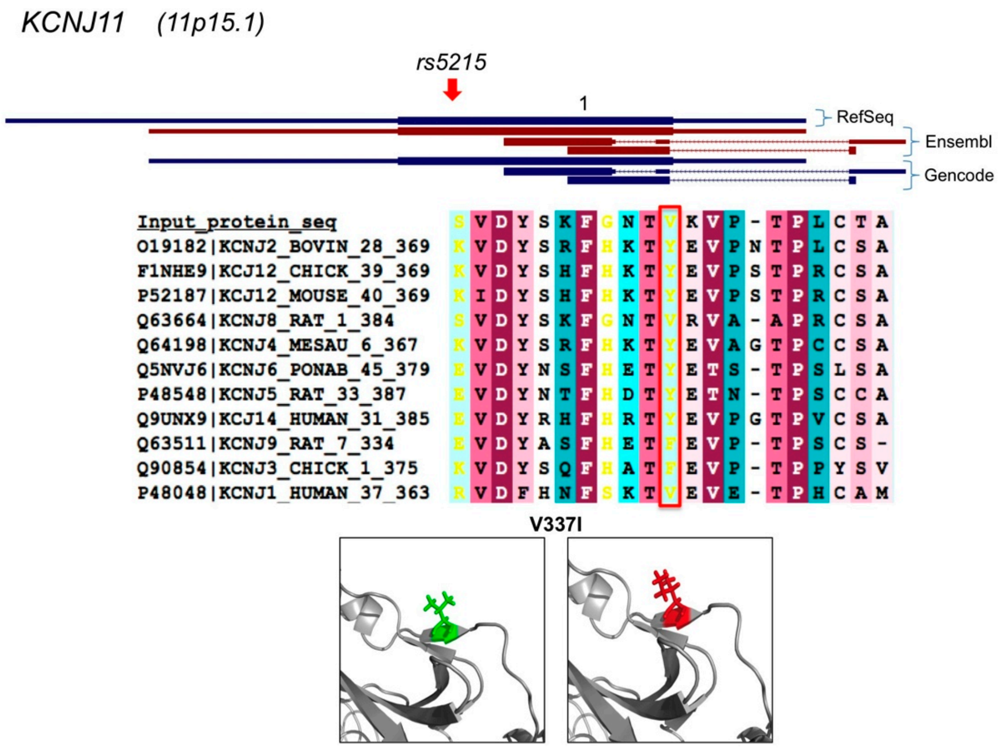

Figure S4. The polymorphism rs5215 consists in a nonsynonimous nucleotide variation G to A that carries to the substitution of a Valine in Isoleucine at the position 250 of protein product. This SNP also overlaps with several genomic features, ranging by TFBS to histone and chromatin modifications, as shown in Figure 2. Moreover, the SIFT and PolyPhen scores predict that such variation may be tolerated. In the multiple sequence alignment, the red square indicates the polymorphic amino acid. In the tridimensional protein structure, the wild-type residue is shown in green, the polymorphic residue in red.

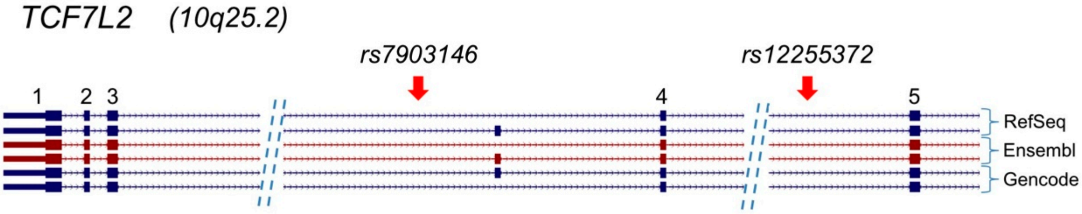

Figure S5. Since both SNPs within the locus *TCF7L2* (RefSeq NM_001146286) do not fall in a coding region, they have not effects on the protein product. Specifically, the G to T substitution annotated as rs12255372 falls within the fourth intron of the gene whereas the C to T substitution, annotated as rs7903146 falls within the third exon. Although these variations do not affect the protein product, we observed that the former falls within the binding sequence of the transcription factor CTCF. However, both rs12255372 and rs7903146 fall respectively within two unspliced EST annotated as AW886557 and T81366 as well as in different histone modification and chromatin remodeling sites (Figure 2). Therefore, these substitutions could have relevant regulatory effects.

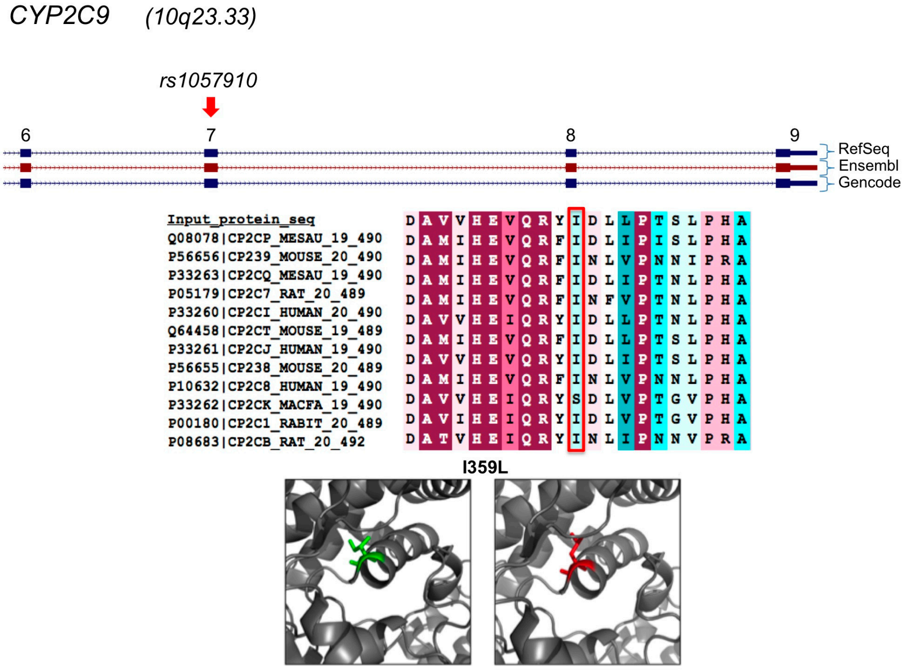

Figure S6. The A/C substitution in the exon 7 of *CYP2C9* gene is predicted to have no significant effects on protein structure and function, since both amino acids—isoleucine and leucine—have similar chemo-physical characteristics and such variation is considered "tolerated" according to SIFT score. In the multiple sequence alignment, the red square indicates the polymorphic amino acid. In the tridimensional protein structure, the wild-type residue is shown in green, the polymorphic residue in red.

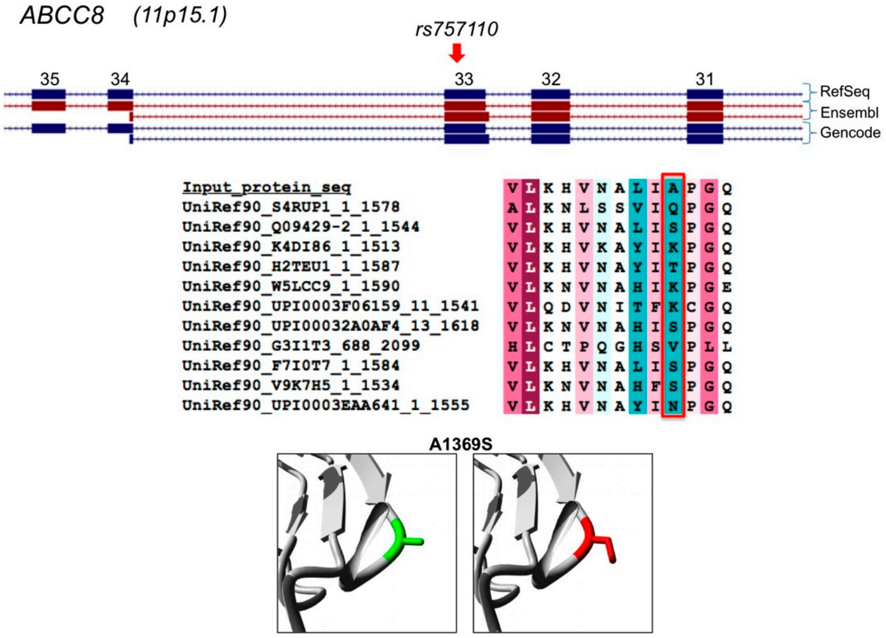

Figure S7. In *ABCC8* gene (RefSeq NM_000352.4), we characterized the SNP rs757110, which causes the substitution of Ala with a Ser residue in position 1369 in the protein (UniProt Q09428). The amino acidic change falls into the cytoplasmic domain ABC transporter 2, which could modify or loss its function. This change is very conserved overall the evolutionary scale (red box). As shown in the figure above, several sequences carry this change at this position. These findings, as well as SIFT and PolyPhen scores, suggest that this SNP may not affect markedly the protein product. In the tridimensional protein structure, the wild-type residue is shown in green, the polymorphic residue in red.

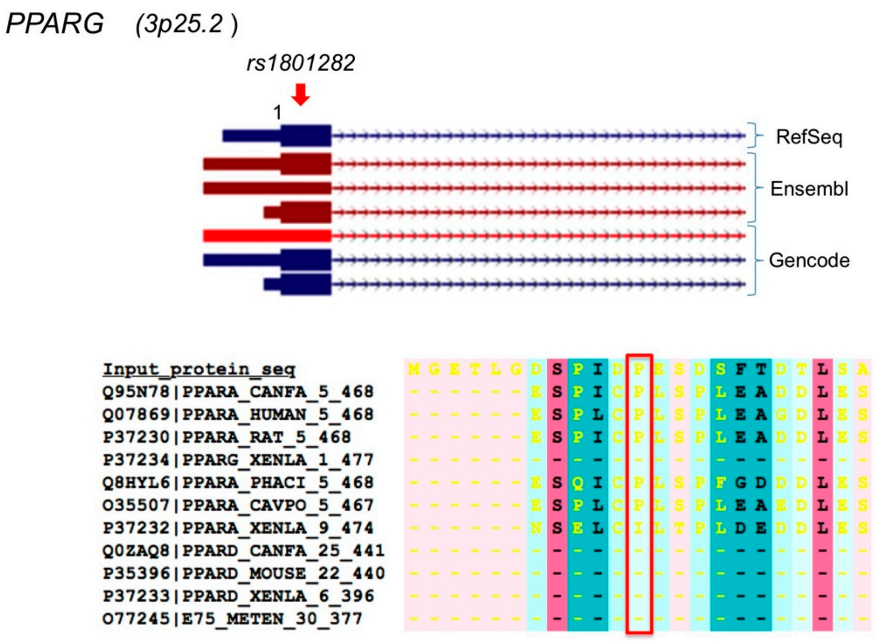

Figure S8. One of the most studied variations in *PPARG* locus (RefSeq NM_015869.4) is rs1801282, which causes the presence, at position 12, of an Ala instead of a Pro in the protein product (UniProt P37231). This change causes a wide range of effects on the transcription factor structure, strongly affecting its function. Reasonably, this variation is not conserved in the evolution and there are not sequences that carry the same substitution (red box). As known, Pro residues induce a local rigidity of the structure, forcing the backbone in a specific conformation. Therefore, this amino acidic substitution is susceptible to disrupt the local structure.

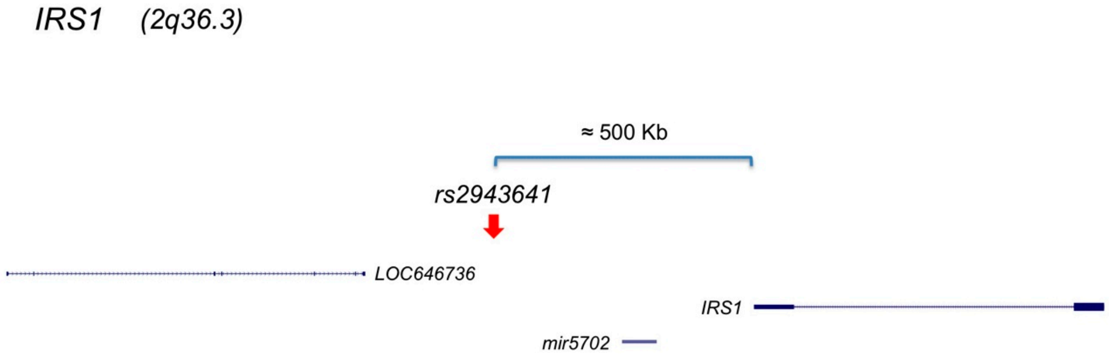

Figure S9. The intergenic SNP annotated as rs2943641 has been analyzed. This substitution falls ≈500 kb upstream the locus. At genomic level, it is located between the long non-coding RNA annotated as *LOC646736* and a microRNA (*MIR5702*). Although no other genomic features have been detected and the considerable distance by *IRS1* gene (RefSeq NM_005544), it could have some effects on transcription regulation, since it overlaps with a CTCF binding site.

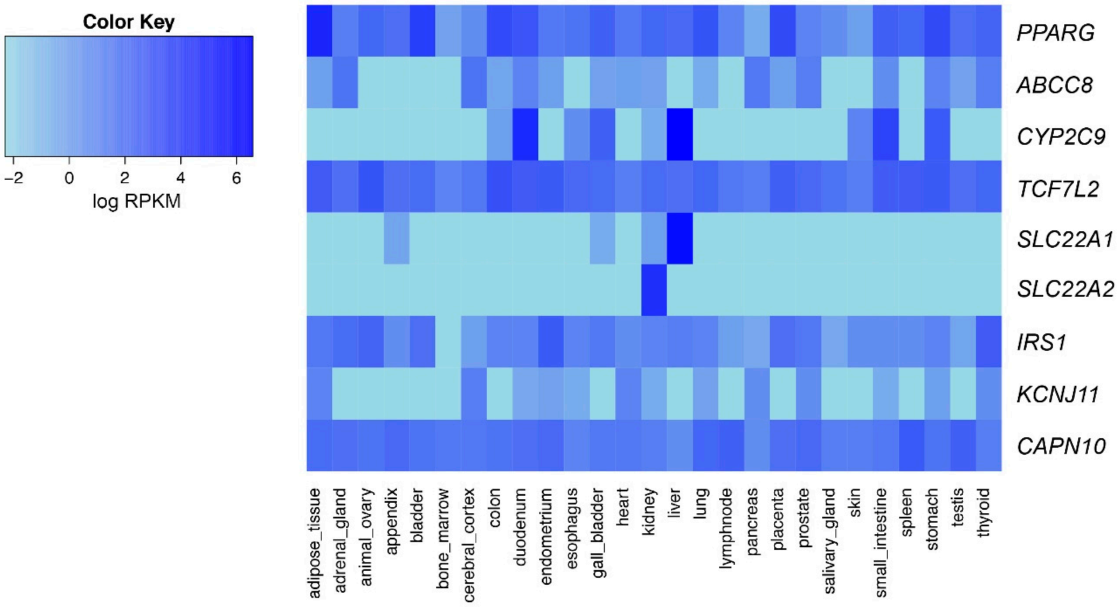

Figure S10. Expression panel of genes responsible for T2D associated drug resistance. The panel shows the normalized expression values of analyzed genes across multiple tissues retrieved from Gene Expression Atlas [15].

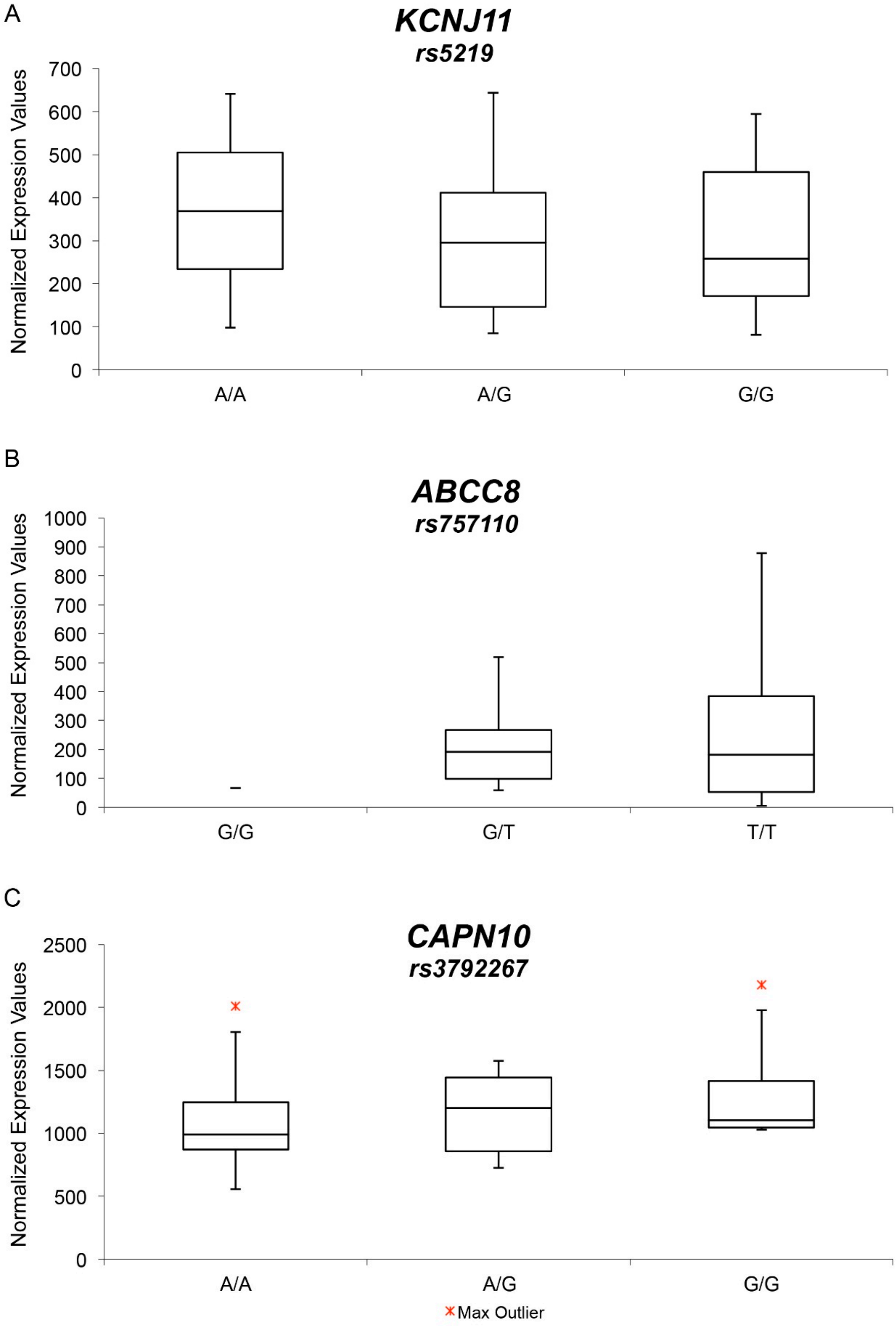

Figure S11. Normalized expression values of wild type, hetero- and homo-zygous polymorphic KCNJ11, ABCC8 and CAPN10 genes. Boxplots show the effect of polymorphisms (in hetero- and homo-zygousity) on the expression of *KCNJ11* (**A**), *ABCC8* (**B**) and *CAPN10* (**C**) genes compared to wild-type counterpart.

Table S1. Cell- and tissue-specific Expressed Sequence Tags and Transcription Factors Binding Sites surrounding the polymorphic sites.

Chromosome	Position	Gene Symbol	SNP ID	Amino Acid Change	Alleles	EST Spliced ID (Tissue)	EST Unspliced ID (Tissue)	Transcription Factor Binding Sites (TFBS) in K562 Cell Line	Transcription Factor Binding Sites (TFBS) in H1-hESC Cell Line	Transcription Factor Binding Sites (TFBS) in GM12878 Cell Line
6	160543148	*SLC22A1*	rs12208357	R61C	C/T	N/A	N/A	FOS-GATA2	CTCF	N/A
	160560824		rs34130495	G401S	A/G	N/A	BX427834 (fetal liver)-BX113941 (n/a)-CF994710 (human placenta)-CA306061(alveolar macrophage)	TFAP2C-MAX-TCF12-TFAP2A-FOS	N/A	N/A
	160560881		rs35167514	420del	-/A	N/A	N/A	MAX-JunB-CTCF	N/A	N/A
	160575837		rs34059508	G465R	A/C/G	BX427834 (fetal liver)-BX11394 (fetal liver spleen)-AA70201 (fetal liver spleen)-CF994710 (human placenta)-AI033457 (n/a)-AW612365 (n/a)	BX427834 (fetal liver)-BX113941 (fetal liver spleen)-AA702013 (fetal liver spleen)-CF994710 (human placenta)-AI033457 (n/a)-AW612365 (n/a)	GATA2-FOS	N/A	N/A
6	160670282	*SLC22A2*	rs316019	S270A	G/T	BX389518 (placenta)-AL544105 (placenta)-BI761325 (n/a)	BX389518-AI306441 (tumor)-AL544105 (placenta)-BI761325-BG899326 (cartilage)	CTCF	N/A	N/A
11	17409572	*KCNJ11*	rs5219	K23E	C/T	BQ917358 (sciatic nerve)	AI792302 (n/a)	HDAC8	CTCF	N/A
	17408630		rs5215	V337I	G/A	BM546966 (n/a)	DC305775 (cerebellum)-CB112941 (n/a)-BE159475 (n/a)-BF842095 (n/a)-DA495241 (brain)	NR4A1	CTCF	CTCF
10	114808902	*TCF7L2*	rs12255372	N/A	G/T	N/A	AW886557(n/a)—AW886568 (n/a)	N/A	N/A	N/A
	114758349		rs7903146	N/A	C/T	N/A	T81366 (fetal liver spleen)	N/A	N/A	N/A
10	96702047	*CYP2C9*	rs1799853	R144C	C/T	various	various	N/A	N/A	N/A
	96741053		rs1057910	I359L	A/C	various	various	N/A	N/A	N/A
11	17418477	*ABCC8*	rs757110	A1369S	G/T	various	DA132773 (alzheimer cortex)-BQ477829 (insulinoma)-BE670869 (carcinoid)-BM263435 (purified pancreatic islet)-R85563 (n/a)-H41529 (n/a)	N/A	cMyc	CTCF
3	12393125	*PPARG*	rs1801282	P12A	C/G	BI820841 (n/a)	BI820841	CTCF	N/A	N/A
2	227093745	*IRS1*	rs2943641	N/A	C/T	N/A	N/A	CTCF	N/A	N/A
	227660544		rs1801278	G971R	G/A	AA128603 (pancreas)	various	N/A	N/A	N/A
2	241534293	*CAPN10*	rs3842570	N/A	I/D	N/A	BF528146 (anaplastic oligodendroglioma)	N/A	N/A	N/A
	241531174		rs3792267	N/A	A/G	N/A	BQ899318 (sciatic nerve)-AL703891 (n/a)-DA094595 (cerebellum)	GATA2	Nr4a1	N/A
	241542703		rs5030952	N/A	C/T	N/A		FOS-CTCF	N/A	N/A
